# When Drivers Step Off the Bus: Well-Being and Turnover Intention in the Public Transport Sector

**DOI:** 10.3390/ijerph23040485

**Published:** 2026-04-12

**Authors:** Diana Carbone, Andrea Colabucci, Francesco Marcatto

**Affiliations:** 1 Department of Life Sciences, University of Trieste, 34128 Trieste, Italy; diana.carbone@units.it; 2 Department of Humanities, University of Trieste, 34128 Trieste, Italy; andrea.colabucci@studenti.units.it

**Keywords:** turnover intention, occupational health, public transportation sector, Job Demands–Resources model, employee retention

## Abstract

**Highlights:**

**Public health relevance—How does this work relate to a public health issue?**

- Public transport is a high-stress sector facing workforce shortages and high turnover rates, making it essential to identify psychosocial factors associated with employee well-being and retention.
- High employee turnover in public transport can negatively affect service reliability, operational safety, and access to safe and sustainable transportation systems, with potential consequences for public health.

**Public health significance—Why is this work of significance to public health?**

- Using the Job Demands–Resources framework, the study identifies two distinct employee well-being profiles characterized by different combinations of stress, engagement, and meaning of work.
- Motivational resources, including satisfaction with pay and the nature of work (i.e., job content and tasks), predicted well-being profile membership, which in turn was associated with turnover intention. Additionally, satisfaction with supervision, perceived workplace safety, and age showed direct associations with turnover intention.

**Public health implications—What are the key implications or messages for practitioners, policy makers and/or researchers in public health?**

- Strengthening supervisory support and improving workplace safety may help reduce turnover intention and support employee retention in the public transport sector, contributing to safer and more reliable mobility systems.
- Resource-oriented interventions, such as fair compensation policies and professional development opportunities, may foster employee well-being and workforce stability, with potential benefits for service continuity, public safety, and environmental sustainability.

**Abstract:**

Voluntary turnover represents a critical challenge in essential public services, where workforce attrition affects both employee well-being and service quality. The primary objective of this study was to identify the psychosocial predictors of well-being profiles and turnover intention among public transport workers, using the Job Demands–Resources model as a theoretical framework. A cross-sectional study design was employed, with 131 employees of an Italian public transport company completing a questionnaire assessing turnover intention and key psychosocial factors (job satisfaction, perceived work-related stress, work engagement, meaning of work, and perceived workplace safety). The analytical strategy integrated Latent Profile Analysis (LPA), logistic regression, and path analysis. LPA identified two distinct well-being profiles: a “low well-being profile,” with high perceived stress and low engagement and meaning of work; and a “high well-being profile,” with low stress and high engagement and work meaning. Logistic regression analyses showed that satisfaction with pay and the intrinsic nature of work tasks predicted membership in the high well-being profile. Path analysis indicated that profile membership significantly predicted turnover intention, with employees in the high well-being profile reporting lower turnover intention. Additionally, satisfaction with supervision, perceived workplace safety, and age showed direct effects on turnover intention. These findings highlight the organizational and psychological resources that can increase employee well-being and retention in the public transport sector, offering insights for preventive interventions and for promoting safer and more sustainable public transport systems.

## 1. Introduction

The term voluntary turnover describes an employee’s decision to voluntarily end their work relationship. Organizations devote considerable attention to turnover due to the strategic importance of human capital and the high costs of replacing valuable people [1,2]. Furthermore, turnover can damage the organization’s reputation [3] and undermine the performance, morale, and productivity of the remaining employees [4]. High turnover rates may also lead to work overload and expanded responsibilities among remaining employees, driven by staff shortages or insufficiently qualified personnel [5], thereby compromising overall organizational well-being.

Turnover intention is defined as an employee’s conscious and deliberate willfulness to leave the organization, and represents the final stage in the withdrawal process, where an individual evaluates the possibility of quitting their current job [6]. As such, it is widely recognized as the best predictor of actual turnover [6], and it has been studied extensively across different organizational sectors. In fact as Lannoo and Verhofstadt point out [7], although unifying the literature on turnover intention is critical to the advancement of knowledge about the phenomenon, the dynamics of the job and the associated processes can vary greatly from sector to sector. For this reason, general models should be supplemented with industry-specific research, in order to enable the development of effective retention policies tailored to each job sector [8].

Among all sectors, public transportation is a particularly complex industry, characterized by worker shortages [9], high job stress [10], unsafe working conditions [11], shift work and related sleep problems [12], and high turnover rates [7]. In this context, high turnover generates a range of critical consequences that negatively affect operations, service quality, safety, and the economic sustainability of companies. For instance, staff shortages may lead to service disruptions and cancellations, increased driving risks due to time pressure, loss of expertise following the departure of experienced drivers, and a heightened risk of burnout among remaining employees [13,14,15]. Moreover, reduced service reliability may encourage greater use of private vehicles, with implications for traffic congestion and environmental emissions [14]. These consequences not only undermine the well-being of workers but also affect service users and the broader community, positioning employee well-being and retention in public transport as a relevant public health issue, particularly in relation to safety, service accessibility, and environmental sustainability. Within this broader context, examining the psychosocial working conditions that shape employee well-being and retention is particularly relevant in national settings where structural and demographic challenges further intensify these risks. In Italy, local public transport employees, including bus, tram, and metro drivers, as well as other operational staff, are covered by the National Collective Labor Agreement (CCNL Autoferrotranvieri Internavigatori), last renewed in 2024. To ensure the continuous availability of transportation services, the typical workweek consists of 39 h, usually structured in irregular shifts that include nights, weekends, and public holidays. The occupation is widely recognized as demanding and high stress, given the responsibility for passenger safety, frequent exposure to hazards, and occasional violent incidents. Empirical evidence in Italian public transport employees indicates that transport workers report higher work pressure and lower mental and physical health and job satisfaction compared with normative samples [16]. Taken together, these psychosocial factors may compromise occupational well-being and reduce the sector’s attractiveness to younger workers. In combination with an aging workforce—about 50% of employees are over 50 years old—this has contributed to chronic understaffing [17]. Despite these structural challenges, empirical research examining the psychosocial determinants of turnover intention in this sector remains limited. To address this gap, the present study adopted a person-centered approach to identify distinct configurations of organizational well-being (i.e., well-being profiles) and to examine how these profiles, along with specific job demands and resources, relate to turnover intention in a public transport company in northeastern Italy.

### 1.1. Antecedents of Turnover Intention

Turnover intention can be influenced by multiple organizational, individual and contextual factors. Job satisfaction is one of the most extensively studied variables that has been proven to be most associated with turnover intention [18,19]. Specifically, job satisfaction appears to serve as a protective factor against turnover intention, while dissatisfaction tends to increase the likelihood of seeking alternative employment [20,21]. Job satisfaction, moreover, has been linked to work engagement, another key antecedent of turnover intention. Several dimensions of job satisfaction have been positively associated with work engagement [22] and have been shown to mediate the relationship between other organizational variables and work engagement [23]. While there is ongoing debate regarding the causal direction between satisfaction and engagement, several studies support the view that positive evaluation of work (satisfaction) precedes the activation of motivation and commitment [24,25,26,27].

Another important antecedent of turnover intention is work-related stress. In fact, stressful working conditions, such as high workloads, time pressure, or role conflict, can lead to lower organizational commitment and to a higher desire to quit [28,29,30,31]. Moreover, as for work engagement, job stress has been frequently associated with reduced levels of job satisfaction [32,33].

Lastly, motivational factors, such as meaningful work, can also influence employees’ withdrawal decisions [34,35]. Meaningful work reflects the subjective perception of work as a source of personal significance, purpose and value in one’s life [36]. In other words, it captures the extent to which work contributes to personal fulfillment and identity. Several studies and theoretical frameworks suggest that this construct is related to various components of job satisfaction, including relationship with colleagues [37], with supervisors [38], and financial rewards [39].

Overall, the constructs of job satisfaction, work engagement, occupational stress, and meaningful work all appear to be relevant predictors of turnover intention. Specifically, literature suggests that job satisfaction may have an antecedent role, influencing the other factors and shaping employees’ decision to stay or leave. Importantly, these factors may not operate in isolation but can co-occur and reinforce one another in shaping employees’ well-being and withdrawal cognitions. In this framework, employee well-being is conceptualized as a multidimensional construct that encompasses both the absence of negative states, such as occupational stress, and the presence of positive psychological functioning and fulfillment, such as work engagement and meaning of work [40]. To provide a coherent theoretical account of how these factors jointly relate to well-being and turnover intention, the present study draws on the Job Demands–Resources (JD-R) model [26,41,42]. According to the JD-R model, job demands (such as high workload, time pressure, and exposure to stressful or unsafe situations) require sustained physical or psychological effort and may lead to strain and health impairment. Conversely, job resources (including supportive supervision, fair pay, meaningful work, and a satisfying nature of work, referring to the intrinsic interest and variety of daily tasks) foster motivation, enhance well-being, and buffer the negative effects of demands. In relation to turnover, prolonged exposure to high job demands may lead to exhaustion and burnout, thereby increasing employees’ intention to leave. Job resources, in contrast, can play a protective role by strengthening work motivation and organizational attachment.

While the JD-R model encompasses a broad range of job demands (e.g., workload, time pressure) and job resources (e.g., autonomy, job control), the present study adopts a context-driven and selective operationalization of the framework. Specifically, job satisfaction, work engagement, and meaningful work can be conceptualized as general key resources, while two additional factors may be particularly relevant and framed as transportation sector-specific demands, beyond general perceived stress: work safety and sleep disturbances linked to shift work. Indeed, both irregular shifts and exposure to workplace violence are known to be associated with sleep problems [43], which in turn may lead to dysfunctional organizational outcomes, such as deviant workplace behaviors [44] and increased turnover intention [45,46]. Accordingly, by selecting a specific set of demands and resources, this study does not aim to provide a comprehensive test of the JD-R model, but rather to examine how a theoretically grounded yet contextually relevant configuration of factors relates to employee well-being and intention to leave in this specific work setting.

Moreover, this framework suggests that these different constellations of job demands and resources may characterize employees in distinct ways, with potential implications for their well-being and intention to leave. From this perspective, adopting a person-centered approach may provide a more refined understanding of turnover processes in this sector.

### 1.2. Objectives

Recent studies on turnover intention in various organizational sectors have increasingly adopted mixture modeling techniques, such as latent profile analysis (LPA), applied to cross-sectional data [47,48,49]. These techniques group individuals with similar patterns of characteristics, such as attitudes, behaviors, or personality traits, allowing researchers to identify homogeneous subgroups and examine how these profiles are related to relevant outcomes [50]. In line with the JD-R model, the present study applied LPA to identify distinct employee subgroups based on sector- specific demands and resources discussed above.

The primary aim of this study was therefore to adopt a person-centered approach to examine how different configurations of psychosocial working conditions (well-being profiles) are associated with turnover intention. The second objective was to identify the key organizational and psychological factors associated with profile membership and turnover intention, with the aim of developing a parsimonious explanatory model. Such a model may contribute not only to theoretical understanding but also to the design of occupational and public health promotion strategies and retention policies, ultimately supporting workers’ well-being, service quality, and environmental sustainability in the public transport sector.

## 2. Materials and Methods

### 2.1. Participants and Procedure

This study employed a cross-sectional design and was approved by the Ethics Committee of the University of Trieste. Bus drivers of a public transportation company in northeastern Italy were invited to take part in the study. Data collection was conducted following formal authorization from the organization. The study was introduced internally through organizational channels, and employees were invited to participate voluntarily by accessing an online questionnaire. On 20 March 2025, they received a link to an online questionnaire, which remained accessible until 30 March 2025. The first page of the questionnaire included written informed consent, specifying that participation was voluntary, that participants could withdraw at any time without providing a reason, and that all measurement instruments were anonymous, with only aggregated results shared with the company. Participants had to click “I consent” to proceed with the study.

Of the 210 employees who received the link, 135 completed the questionnaire, resulting in a 64% response rate. Four respondents were excluded due to excessive missing values (>40%), resulting in a final sample of 131 employees.

### 2.2. Measures

Job Satisfaction. The Job Satisfaction Survey (JSS) [51] was used to assess job satisfaction. This questionnaire is composed of 36 items that cover nine facets of job satisfaction: pay (e.g., “*I feel I am being paid a fair amount for the work I do*”), promotion (e.g., “*I am satisfied with my chances for promotion*”), supervision (e.g., “*My supervisor is unfair to me*”), fringe benefits (e.g., “*I am not satisfied with the benefits I receive*”), contingent rewards (e.g., “*I do not feel that the work I do is appreciated*”), operating conditions (e.g., “*I have too much to do at work*”), co-workers (e.g., “*I enjoy my coworkers*”), nature of work (e.g., “*I like doing the things I do at work*”), and communication (e.g., “*Communications seem good within this organization*”). Items are rated on a 6-points Likert scale, ranging from 1 (“strongly disagree”) to 6 (“strongly agree”). Each subscale score is obtained by summing its four items, with higher scores indicating greater satisfaction. In this study, Cronbach’s alpha values for the subscales ranged from 0.71 to 0.86, except for the Operating Conditions subscale, which yielded an alpha of 0.41. This low reliability likely reflects the presence of items more relevant to office-based roles than to the operational environment examined in this research. Consequently, the subscale was excluded from further analyses.

Work-related stress. The Perceived Occupational Stress scale (POS) [52] was employed to measure the level of stress experienced at work. A 5-point Likert scale is used to rate the scale’s four items (e.g., “*At work I feel under pressure*”), from 1 (“strongly disagree”) to 5 (“strongly agree”). The total score is the average of item responses, with higher scores indicating a greater level of occupational stress. Cronbach’s alpha in the current study was 0.87.

Work engagement. To assess work engagement, the short version of the Utrecht Work Engagement Scale (UWES-9) [53] was employed, which is composed of nine items (e.g., “*I am immersed in my work*”) rated on a 7-point Likert scale ranging from 0 (“never”) to 6 (“always”). Scores are averaged to compute the work engagement total score, with higher scores denoting greater engagement. Cronbach’s alpha in the current study was 0.95.

Meaning of work. The Work And Meaning Inventory (WAMI) [54] was used to assess the subjective meaning of work. This questionnaire is composed of ten items (e.g., “*I understand how my work contributes to my life’s meaning*”) rated on a 7-point Likert scale ranging from 1 (“strongly disagree”) to 7 (“strongly agree”). The total score is obtained by summing all item responses, with higher scores indicating a greater perceived meaning of work. Cronbach’s alpha in the current study was 0.95.

Shift work disorder. Shift work disorder (SWD) was assessed by two questions adapted from a recent study of sleep disturbances related to shift work [55] and based on the diagnostic criteria of the third edition of the International Classification of Sleep Disorders [56]: “*Do you have a work schedule that sometimes overlaps with the time you usually sleep?*”, and “*If yes, does this cause insomnia and/or excessive sleepiness due to reduced amount of sleep?*”. Responses were dichotomous (yes/no). Participants answering “yes” to both questions were classified as having possible SWD.

Work safety. The Job Safety subscale of the Work Safety Scale (WSS) [57,58] was used to assess the perception of workplace safety. This subscale is composed of ten items consisting of descriptive adjectives or short phrases (e.g., “*Dangerous*”) that participants are asked to evaluate in relation to their own job. Responses were rated on a 5-point Likert scale ranging from 1 (“strongly disagree”) to 5 (“strongly agree”). The total score is the average of item responses, with higher scores implying higher perceived safety. Cronbach’s alpha in the current study was 0.95.

Turnover intention. Turnover intention was assessed with the Ordinal Turnover Intention Scale (OTIS) [21] which captures an employee’s current stage in the multi-stage turnover intention process [6]. The OTIS consists of a single item (“*The following statements pertain to your current situation regarding your job. Please select the one that you believe most accurately represents your present condition*”) and participants select one of six ordinal statements, ranging from “*At the moment, I don’t think I should change my current job*” (coded as 0) to “*I am about to voluntarily leave my current job*” (coded as 5).

### 2.3. Data Analysis

First, standard descriptive statistics were computed for turnover intention and all other study variables. To address the potential for common method bias due to the self-reported and cross-sectional nature of the data, Harman’s single-factor test was performed using an unrotated exploratory factor analysis (EFA) including all study variables. The results revealed that the first factor accounted for 37.3% of the total variance, which is below the 50% threshold [59], suggesting that common method bias was not a significant concern for the present study. Subsequently, a LPA was conducted using perceived occupational stress, work engagement, and meaning of work as indicators. Because the three variables were measured on different scales, their scores were standardized into z-scores prior to analysis. Latent profile models were estimated by progressively increasing the number of profiles. Model fit was evaluated using multiple indices: Log-likelihood value, Akaike information criterion (AIC), Bayesian information criteria (BIC), Sample-Size Adjusted Bayesian Information Criterion (SABIC), Integrated Completed Likelihood (ICL), and Entropy. To identify the variables associated with latent profile membership and turnover intention, logistic regression and ordinal logistic regression analyses were conducted separately. Finally, a path analysis was performed using the diagonally weighted least squares (DWLS) estimation method with robust standard errors to evaluate the fit of a model in which turnover intention was predicted by latent profile membership and the variables that emerged as significant predictors in the ordinal logistic regressions. The DWLS method is recommended when dealing with ordinal variables because it provides more accurate parameter estimates and standard errors than methods assuming continuous data [60]. The following fit indices were evaluated: Root Mean Square Error of Approximation (RMSEA), Standardized Root Mean Square Residual (SRMR), Comparative Fit Index (CFI), and Tucker-Lewis Index (TLI). Values lower than 0.06 for RMSEA, lower than 0.08 for SRMR, and higher than 0.95 for CFI and TLI indicate an adequate fit to the data [61]. Given the cross-sectional design of the study, all results should be interpreted as associations rather than causal relationships, and the directionality implied by the statistical models reflects theoretical assumptions rather than empirical causal evidence. All analyses were conducted using Jamovi v2.6.25.

## 3. Results

### 3.1. Descriptive Statistics

Demographic and work-related data of the study sample are reported in Table 1.

Table 2 shows the frequencies of OTIS categories. Nearly half of the participants (46.6%) reported no intention to leave their current job (level 0, “At the moment, I don’t think I should change my current job”). About one third reported to occasionally think about changing jobs (level 1, “Sometimes, I think I should change my current job”), and about 20% of participants were in a more advanced stage of the turnover decision-making process (levels 2–4, from “I am seriously starting to consider the possibility of looking for a new job” to “I have found new job opportunities, and I am seriously evaluating them”). No participant selected level 5 (“I am about to voluntarily leave my current job”).

The descriptive statistics for the other study variables are presented in Table 3. Correlations among measures are reported in Supplementary Material Table S1.

Regarding sleep problems due to shift work, 57 participants (47.9%) had answered yes to both questions and were therefore classified as having a possible work shift disorder.

### 3.2. Latent Profile Analysis

To determine the optimal number of latent profiles based on perceived occupational stress, work engagement, and meaning of work, models with two to four latent profiles were estimated, and their fit indices were examined (Table 4). The two-profile solution had the lowest BIC, indicating the best balance between model fit and parsimony. Although the four-profile solution provided a slightly higher log-likelihood, it also yielded a higher BIC and lower entropy than the two-profile model, suggesting reduced classification accuracy. Therefore, the two-profile model was selected as the most appropriate.

**Figure 1:**
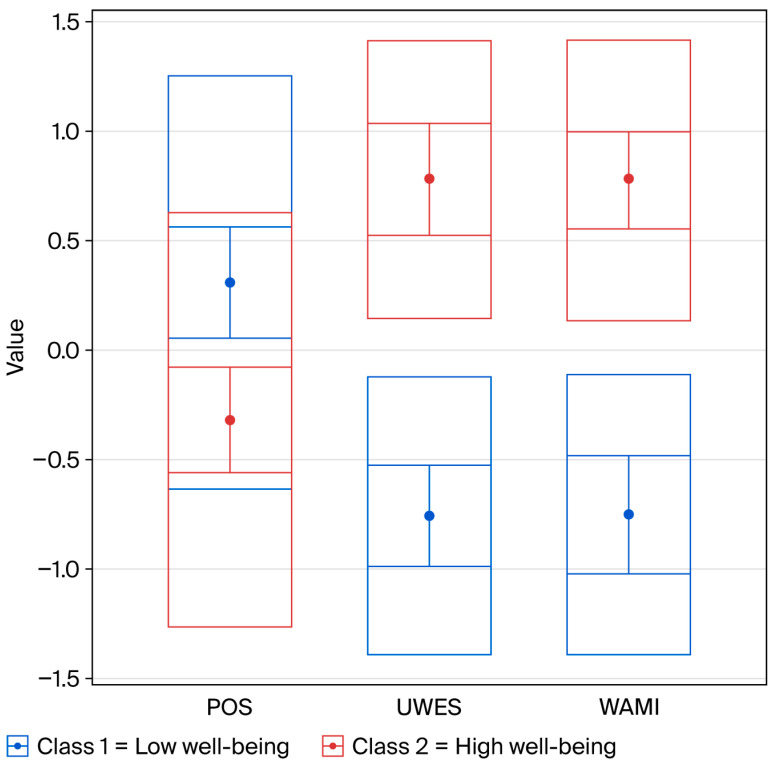
Standardized Scores of Stress, Engagement, and Meaning of Work by Latent Profile. Note. WAMI = The Work And Meaning Inventory; UWES = Utrecht Work Engagement Scale; POS = Perceived Occupational Stress. Class 1 = low well-being, Class 2 = high well-being. Scores are presented as standardized z-scores. Mean standardized indicator values for each latent profile. Inner error bars represent 95% confidence intervals of the mean; outer boxes represent one standard deviation.

Figure 1 shows the distribution of the two profiles across levels of perceived occupational stress (POS), work engagement (UWES-9), and meaning of work (WAMI).

Profile 1, labeled “low well-being profile”, included 50.4% of participants (N = 66) and was characterized by high levels of perceived occupational stress (*M* = 3.14, *SD* = 1.12), along with low levels of work engagement (*M* = 3.12, *SD* = 0.99) and meaning of work (*M* = 30.4, *SD* = 10.05). Conversely Profile 2, labeled “high well-being profile”, included 49.6% of participants (N = 65) and was characterized by low levels of perceived occupational stress (*M* = 2.44, *SD* = 0.96), and high levels of both work engagement (*M* = 5.61, *SD* = 0.89) and meaning of work (*M* = 53.7, *SD* = 9.07). The quality of classification was evaluated using Average Assignment Probabilities (AAP). Participants assigned to the Low well-being profile had a mean probability of 0.941 (*Mdn* = 0.996), while those assigned to the High well-being profile had a mean probability of 0.932 (*Mdn* = 0.997). These values indicate that most participants were classified with high certainty into their respective profiles, confirming the robustness and clarity of the two-profile solution. Next, a Mann-Whitney U test was conducted to investigate differences in turnover intention between the two profiles. Results showed a significant difference between the groups (*U* = 815, *p* < 0.001), with participants in the low well-being profile reporting higher turnover intention (*Mdn* = 1.00) than those in the high well-being profile (*Mdn* = 0.00).

### 3.3. Antecedents of Membership in the Two Profiles

A logistic regression was conducted to identify predictors of membership in the two profiles. This analysis also allowed for establishing the criterion-related validity of the identified latent profiles [50]. Predictors included all the job satisfaction subscales (pay, promotion, supervision, fringe benefits, contingent rewards, co-workers, nature of work, and communication) and work safety. An initial check for multicollinearity revealed that all variance inflation factor (VIF) values were below 5, suggesting no problematic collinearity among the predictors. As reported in Table 5, only the JSS subscales Pay (*b* = 0.21, *SE* = 0.10, *p* = 0.034) and Nature of Work (*b* = 0.46, *SE* = 0.12, *p* < 0.001) significantly predicted profile membership. Specifically, higher scores on these subscales were associated with greater odds of belonging to the “high well-being profile” rather than the “low well-being profile”, with odds ratios of 1.23 (95% CI [1.02, 1.50]) and 1.58 (95% CI [1.26, 1.99]), respectively.

### 3.4. Antecedents of Turnover Intention

An ordinal logistic regression was conducted with turnover intention as the dependent variable. Predictors included the job satisfaction subscales, work safety, work shift disorder, and latent profile membership, controlling also for age, given the expectation that turnover intention would be higher among younger bus drivers. All VIF values were below 5, suggesting no problematic collinearity among predictors. As shown in Table 6, the JSS subscales Supervision (*b* = −0.30, *SE* = 0.07, *p* < 0.001), Fringe Benefits (*b* = −0.22, *SE* = 0.11, *p* = 0.048) and Communication (*b* = 0.18, *SE* = 0.08, *p* = 0.019) significantly predicted turnover intention. Higher satisfaction with supervision and fringe benefits was associated with lower odds of expressing higher turnover intention (OR Supervision = 0.74, 95% CI [0.64, 0.86], *p* < 0.001; OR Fringe Benefits = 0.80, 95% CI [0.65, 0.99], *p* = 0.048). Interestingly, higher satisfaction with organizational communication was associated with increased odds of turnover intention (OR = 1.20, 95% CI [1.03, 1.40], *p* = 0.019). Perceived work safety also significantly predicted turnover intention (*b* = −0.82, *SE* = 0.39, *p* = 0.037), with higher perceived safety linked to lower odds of expressing higher turnover intention (OR = 0.44, 95% CI [0.20, 0.95], *p* = 0.037). Age was another significant predictor (*b* = −0.12, *SE* = 0.03, *p* < 0.001) and, as expected, older bus drivers reported lower odds of higher turnover intention (OR = 0.88, 95% CI [0.82, 0.95], *p* < 0.001). Finally, latent profile membership significantly predicted turnover intention levels (*b* = −2.26, *SE* = 0.65, *p* < 0.001), with membership in the “high well-being profile” associated with a lower probability of reporting higher turnover intention (OR = 0.1, 95% CI [0.03, 0.37]).

### 3.5. Path Analysis

A path analysis was conducted to test an initial model of turnover intention (Model 1), which included all the variables that emerged as significant predictors in the preceding ordinal regression analysis (i.e., Supervision, Communication, Work Safety, Latent Profile Membership, and age) and the predictors of latent profile membership (Pay and Nature of Work). Although Fringe Benefits was marginally significant in the regression model (*p* = 0.048), the corresponding confidence interval of the odds ratio included values very close to the null (OR = 0.80, 95% CI [0.65, 0.998]), suggesting that the effect might be weak or unstable. In line with recommendations to avoid overfitting and to consider both statistical and practical significance when specifying structural models [62], this variable was excluded from the path analysis.

**Figure 2:**
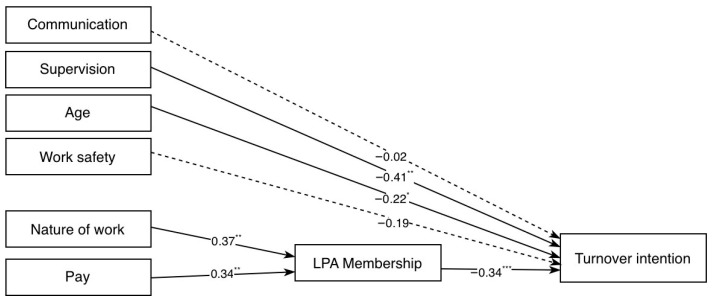
Initial path model of turnover intention (Model 1). Note. Standardized path coefficients are reported. * = *p* < 0.05, ** = *p* < 0.01, and *** = *p* < 0.001. Dotted lines represent non-significant paths.

As shown in Table 7, Model 1 demonstrated excellent fit indices (CFI = 1.00; TLI = 1.03; SRMR = 0.02; RMSEA = 0.00, 90% CI [0.00–0.05]). However, these indices should be interpreted with caution, due to the model’s low complexity and the estimation method used. Indeed, DWLS is known to produce very high fit values in models with simple structures and ordinal data, potentially leading to an overestimation of model fit [60]. Therefore, interpretation of the model should be guided not only by fit indices, but also by theoretical coherence and the statistical significance of the estimated paths. In this model, indeed, not all hypothesized paths were statistically significant. Specifically, as shown in Figure 2, the paths from Work Safety to turnover intention (*b* = −0.17, *SE* = 0.09, *p* = 0.066) and from Communication to turnover intention (*b* = −0.00, *SE* = 0.09, *p* = 0.824) didn’t reach statistical significance.

**Figure 3:**
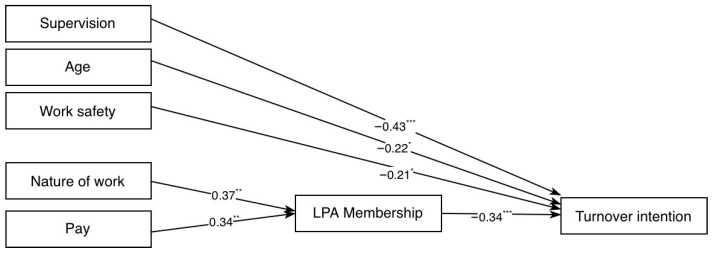
Final path model of turnover intention (Model 2). Note. Standardized path coefficients are reported. * = *p* < 0.05, ** = *p* < 0.01, and *** = *p* < 0.001.

Therefore, a more parsimonious model (Model 2) was tested, in which the weakest non-significant predictor (Communication) was removed. This revised model aimed to determine whether a simpler structure could maintain or improve fit without substantially compromising explanatory power. As shown in Table 7, Model 2 also demonstrated excellent fit, (CFI = 1.00, TLI = 1.02, SRMR = 0.02, and RMSEA = 0.00, 90% CI [0.00, 0.08]), comparable to Model 1 but with improved parsimony. Moreover, as shown in Figure 3, the path from Work Safety to turnover intention reached statistical significance (*b* = −0.19, *SE* = 0.08, *p* = 0.023), thereby strengthening support for this model. For these reasons, Model 2 was retained as the final model.

The squared multiple correlation coefficients (R^2^) indicated that Model 2 accounted for 56% of the variance in turnover intention and 41% of the variance in the latent profile membership. To assess the mediating role of profile membership, indirect effects were tested. Results showed that both Pay (IE_1_ = −0.02, 95% CI [−0.04, −0.004], β = −0.12, *p* = 0.017) and Nature of Work (IE_2_ = −0.03, 95% CI [−0.05, −0.01], β = −0.13, *p* = 0.008) had significant negative indirect effects on turnover intention via latent profile membership.

Although the model specifies directional paths, these should be interpreted as statistical associations rather than causal effects due to the cross-sectional design.

## 4. Discussion

This study aimed at identifying the key organizational and psychological factors associated with well-being profiles and turnover intention in a public transportation company. Although no participant indicated that they were about to leave their job, more than half of the sample reported at least some degree of intention to quit. These findings suggest that, even in the absence of acute withdrawal intentions, turnover considerations are present and represent a relevant organizational concern, with potential implications for service continuity, operational safety, and, more broadly, public health. The relatively low endorsement of extreme responses may partly reflect selection bias (i.e., employees on the verge of leaving may have been less inclined to participate) or social desirability processes, as employees sometimes underreport turnover intentions due to fear of negative consequences [63]. Even low-to-moderate levels of turnover intention, however, should not be underestimated, as they may represent the early stages of psychological withdrawal that, over time, can evolve into actual turnover.

In line with a growing body of research adopting a person-centered perspective on organizational well-being [64,65], the latent profile analysis identified two distinct configurations of psychosocial working conditions. The first profile, labeled “low well-being profile”, was characterized by high levels of perceived occupational stress and low levels of both work engagement and meaning of work. The second, labeled “high well-being profile”, was marked by low levels of perceived occupational stress and high levels of both work engagement and meaning of work. These findings suggest that employees in this sector, at least within the context examined in this study, tend to cluster into clearly differentiated well-being patterns rather than varying only along single isolated dimensions. Importantly, membership in these profiles was significantly associated with turnover intention, with employees in the “low well-being profile” reporting significantly higher levels of intention to quit than those in the “high well-being profile”.

The pattern of results observed in the path analysis provides further insight into the factors associated with turnover intention. Profile membership was strongly associated with turnover intention, with belonging to the “high well-being profile” being associated with a lower likelihood of reporting high levels of turnover intention. Profile membership, in turn, was predicted by satisfaction with pay and with the nature of work, which reflects the extent to which employees find their daily tasks and job content rewarding and stimulating. Satisfaction with supervision, perceived work safety, and age exerted direct effects on turnover intention, independent of profile membership. Taken together, these findings suggest the coexistence of indirect and direct associations, whereby some factors are linked to turnover intention through broader well-being configurations, while others show more immediate associations with withdrawal cognitions.

Satisfaction with pay and with the nature of work emerged as key resources associated with membership in the high well-being profile, which in turn was associated with lower turnover intention. These findings highlight the relevance of both extrinsic and intrinsic motivational components in sustaining employees’ psychological well-being. Consistent with prior research, job satisfaction appears to influence employees’ well-being [66], support work engagement [67], and amplify the positive effects of meaningful work [68]. In the Italian transport context, the salience of pay satisfaction is particularly meaningful given ongoing debate regarding working conditions and contract negotiations in this industry [17].

Satisfaction with supervision showed the strongest association with turnover intention, underscoring the protective role of relational support in employees’ decisions to remain in the organization [69]. Perceived work safety and age also emerged as significant direct predictors of turnover intention. Higher perceived safety at work was associated with lower odds of turnover intention, consistent with previous research indicating that when employees feel physically and psychologically safe in their work environment, they are more likely to stay in their jobs, as safety fosters perceptions of job quality and organizational trust, which are key drivers of retention [70,71,72], especially in high-risk sectors such as transportation [73]. Similarly, age showed a negative association with turnover intention, in line with previous studies indicating lower mobility among older workers [74]. However, workforce aging in transport settings may simultaneously pose operational challenges related to cognitive and visual functioning [75], highlighting a complex interplay between retention and safety considerations.

As for the unexpected positive association between communication and turnover intention observed in the regression analysis, it did not remain stable in the path analysis model, suggesting that this effect may reflect suppression or context-specific dynamics rather than a robust predictor. Indeed, communication showed moderate to strong correlations with all other JSS subscales, suggesting substantial conceptual and empirical overlap. In such conditions, regression coefficients may reflect unstable estimates of unique effects, leading to attenuation or even sign reversal when additional variables are accounted for. Accordingly, communication may not represent an independent correlate of turnover intention in this context, but rather reflects more complex interrelations among organizational factors. A further interpretation may be related to the overlap between communication and supervision. In the present study, satisfaction with supervision emerged as the factor most strongly associated with turnover intention, and it is plausible that this construct also captures relational and communicative aspects of the supervisor–employee interaction. For instance, items reflecting supervisors’ attention to employees’ needs or concerns may implicitly involve communication quality. As a result, the variance associated with communication may be partially absorbed by supervision in multivariate models, contributing to the instability of its effect. Likewise, shift work disorder did not emerge as a significant correlate of turnover intention in the final model, despite its relatively high prevalence in the sample. One possible explanation relates to the nature of the measurement employed, which captured only the presence of sleep-related difficulties, rather than employees’ subjective perception of their impact on well-being. From a theoretical perspective, it is plausible that the relationship between shift work disorder and turnover intention is not direct but rather operates through employees’ appraisal of how such conditions affect their daily functioning and quality of life. In line with stress appraisal frameworks such as the Transactional Model of Stress [76], objective conditions may influence outcomes primarily through their subjective interpretation. Accordingly, future research should consider incorporating measures of perceived impact or strain related to shift work in order to better capture its role in turnover processes. Nevertheless, nearly half of the participants reported a possible work shift disorder, indicating a relevant occupational health concern that, although not related to the well-being and turnover factors investigated in this study, could still compromise workers’ health and safety [77].

Taken together, these findings align with the JD-R framework and reveal the coexistence of indirect and direct pathways associated with turnover intention. While certain resources showed indirect associations through broader well-being configurations, others revealed direct associations with withdrawal cognitions. Notably, the overall pattern suggests that the motivational resources were more strongly associated with turnover intention than the examined demands in this context. The divergence between our findings and those of recent research [14], which emphasized the predominant role of demands, may be attributed to contextual differences in the transport sector. While previous studies often focused on high-intensity urban environments with extreme traffic congestion and strict scheduling (heavy demands), our sample may have perceived organizational resources, such as supervisor support and pay satisfaction, as more salient due to the specific socio-economic climate of the Italian transport industry. In contexts where job security is relatively high but wage stagnation is a concern, the motivational process of the JD-R framework (driven by resources) might override the impairment process (driven by demands) in shaping the decision to stay or leave. This finding is particularly relevant for both practitioners, corporate management, and public health stakeholders as it suggests that organizational and relational resources may be more amenable to intervention than sector-specific demands. While structural demands, such as rigid shift schedules, traffic congestion, and the inherent risks of urban driving, are often difficult to reduce or eliminate, management has a greater degree of control over improving supervisory support, refining compensation systems, and fostering a positive safety climate. Therefore, focusing on resource enhancement may represent a more feasible and effective pathway for improving employee retention in the public transport sector.

### 4.1. Implications for Occupational Health Promotion

From an occupational health promotion perspective, these findings underscore the potential of resource-oriented interventions to enhance employee retention in public transport. In particular, positive relationships with supervisors and effective work safety management appear to be strongly associated with bus drivers’ willingness to stay with the company. Strengthening leadership practices could be achieved through targeted training for supervisors focusing on relational leadership and supportive communication. Such training should aim to improve supervisors’ ability to provide constructive feedback, recognize individual effort, and offer emotional support during high-stress situations, such as traffic accidents or service disruptions. Regarding work safety, effective initiatives should move beyond mere technical compliance to foster a proactive safety climate. This includes implementing anonymous “near-miss” reporting systems, where drivers can report hazards without fear of retribution, and providing specific training on de-escalation techniques to manage physical or verbal aggression from passengers, a significant source of perceived risk in the transport sector. These measures represent concrete strategies not only to enhance employee retention but also to promote safer and more sustainable transport services, which are relevant from a public health perspective. Although age is a demographic variable, it provides a strategic insight, namely the importance of focusing retention efforts on younger employees, who are more likely to consider leaving. At the same time, workforce planning should consider the complex balance between retention and safety in aging transport workers, as cognitive and visual changes associated with age may have operational implications.

Consistent with the central role of job-related resources identified in the model (i.e., satisfaction with pay and nature of work), interventions such as fair compensation policies, performance-related bonuses, and opportunities for professional development may foster higher well-being profiles and reduce withdrawal intentions. From an occupational health promotion perspective, strengthening such resources may represent a preventive strategy, mitigating risks of burnout, unsafe behaviors, and service disruptions.

Finally, the person-centered approach adopted in this study suggests that retention initiatives should combine systemic interventions, such as implementing transparent career progression pathways, revising company-wide compensation policies, and establishing organizational-level safety management systems, with targeted actions tailored to the experiences and needs of different employee groups. By embedding these resources into the organizational culture, companies can ensure a more stable and supportive environment for all staff. Considering the broader societal implications of public transport, including passenger safety, service continuity, and environmental sustainability, investing in psychosocial work environment improvements may generate benefits that extend beyond the organization itself, contributing to the overall quality and reliability of essential urban mobility services.

### 4.2. Limitations

Although the study presents notable strengths, including the combined use of a person-centered (LPA) and a variable-centered approach (path analysis), which provided a more detailed understanding of how individual experiences and job-related factors are associated with turnover intention, several limitations should be acknowledged. First, the context-specific nature of the sample, while reducing potential variability due to inter-company differences, limits the generalizability of the findings to other companies, regions, or national settings. Replication across multiple organizations and countries would be necessary to assess the robustness and broader applicability of the proposed model. Second, although the study was conducted anonymously, potential selection bias, the influence of social desirability and fear of retaliation may have affected the response rate and led to an underestimation of actual turnover intentions among respondents. Third, some theoretically relevant job demands and resources—such as perceived workload and job control—were not included in this study. Their omission limits the comprehensiveness of the JD-R operationalization, and future research should incorporate a broader set of psychosocial variables to better capture the dynamics linking work environment, well-being, and turnover intention. Fourth, an additional limitation concerns the use of a single-item measure (OTIS) to assess turnover intention. Although this instrument has been previously validated and is suitable for capturing the progressive nature of turnover intention [21], single-item measures may be more susceptible to measurement error and may not fully capture the complexity of the underlying construct. This issue is particularly relevant in the context of the present analytical strategy, which includes multivariate and path modeling approaches, as measurement limitations may influence the strength and stability of the observed associations. Future research could benefit from the use of multi-item measures to provide a more comprehensive assessment of turnover intention. Finally, the cross-sectional nature of the study precludes causal inferences, and longitudinal research is needed to clarify the directionality of the observed relationships.

## 5. Conclusions

This study contributes to the understanding of employees’ well-being and voluntary turnover dynamics by applying a person-centered perspective grounded in the JD-R framework within a specific organizational context in the public transportation sector. In this study, well-being is conceptualized as a multidimensional construct encompassing both the absence of negative states (e.g., occupational stress) and the presence of positive psychological functioning (e.g., work engagement and meaning of work). Overall, the findings suggest that, within this organizational context, job-related resources, particularly supervisory support and work safety, are more strongly associated with turnover intention than the demands examined, indicating that strengthening existing organizational resources may represent a practical and impactful strategy for employee retention. Although derived from a single organizational context, these findings may be particularly relevant in light of current workforce challenges in the transportation industry. Across Europe and beyond, public transportation companies are struggling to recruit and retain qualified personnel, especially in operational roles such as bus driving. In this context of labor shortages and high job demands, understanding and addressing the early indicators of job withdrawal is critical. Furthermore, turnover problems in the public transport sector may have consequences that extend beyond the organizational level, including service disruptions, safety risks, traffic congestion, and environmental impacts, underscoring the broader societal relevance of workforce stability in essential transport services. Promoting employees’ well-being in essential public services therefore represents not only an organizational priority but also a matter of occupational and public health. Given the relatively small, single-organization sample, these findings should be interpreted with caution and may not fully generalize to other public transport settings. Nevertheless, they highlight the need for further research and targeted interventions aimed at strengthening workforce retention and sustaining the social and environmental functions of public transportation systems.

## Figures and Tables

**Table 1:** Demographic and Work-Related Characteristics of the Study Sample.

Variables	*n* (%)/Mean (SD)
Gender	
Female	16 (12.2%)
Male	115 (87.8%)
Age (years, range 28–61)	47.5 (7.59)
Years of job experience	
0–1	7 (5.3%)
2–5	24 (18.3%)
6–10	17 (13.0%)
11–20	32 (24.4%)
21–30	39 (29.8%)
>30	12 (9.2%)

**Table 2:** Frequencies of OTIS categories.

OTIS		Frequency (%)
#	Category Label	
0	At the moment, I don’t think I should change my current job	61 (46.6%)
1	Sometimes, I think I should change my current job	44 (33.6%)
2	I am seriously starting to consider the possibility of looking for a new job	20 (15.3%)
3	I am actively looking for a new job	2 (1.5%)
4	I have found new job opportunities and am seriously evaluating them	4 (3.1%)
5	I am about to voluntarily leave my current job	0

**Table 3:** Descriptive statistics of the measures of interest.

Variables	Min/Max	Mean (SD)
Pay (JSS)	4/24	13.81 (4.46)
Promotion (JSS)	4/20	10.85 (4.24)
Supervision (JSS)	4/24	16.77 (4.74)
Fringe Benefits (JSS)	4/24	12.23 (4.28)
Contingent Rewards (JSS)	4/24	12.78 (5.17)
Coworkers (JSS)	4/24	16.38 (4.30)
Nature of Work (JSS)	4/24	17.56 (4.09)
Communication (JSS)	4/24	14.81 (5.01)
Job Safety (WSS)	1/5	3.32 (1.07)
WAMI	10/70	41.96 (15.12)
UWES-9	1/7	4.36 (1.56)
POS	1/5	2.79 (1.10)

Note. JSS = Job Satisfaction Scale; WSS = Work Safety Scale; WAMI = The Work And Meaning Inventory; UWES-9 = Utrecht Work Engagement Scale; POS = Perceived Occupational Stress.

**Table 4:** Fit indexes of Latent Profiles model.

Model	LogLik	AIC	BIC	SABIC	ICL	Entropy
2 profiles	−471.05	968.11	1005.03	963.94	−1016.75	0.850
3 profiles	−475.59	987.18	1035.77	981.85	−1089.44	0.616
4 profiles	−468.71	973.42	1025.17	968.24	−1063.33	0.798

Note. LogLik = Log-likelihood value. AIC = Akaike information criterion. BIC = Bayesian information criteria; SABIC = Sample-Size Adjusted Bayesian Information Criterion; ICL = Integrated Completed Likelihood.

**Table 5:** Predictors of the latent profile membership.

Predictors	Latent Profile Membership			
	*b*	SE	*p*	OR (95% CI)
Pay (JSS)	0.21	0.10	0.034	1.23 * (1.02–1.50)
Promotion (JSS)	0.03	0.09	0.790	1.02 (0.85–1.23)
Supervision (JSS)	−0.04	0.09	0.676	0.96 (0.81–1.14)
Fringe Benefits (JSS)	0.13	0.10	0.204	1.14 (0.93–1.40)
Contingent Rewards (JSS)	−0.05	0.12	0.641	0.95 (0.75–1.19)
Coworkers (JSS)	−0.04	0.08	0.556	0.96 (0.82–1.11)
Nature of work (JSS)	0.46	0.12	<0.001	1.58 *** (1.26–1.99)
Communication (JSS)	−0.05	0.08	0.578	0.95 (0.81–1.13)
Work safety	0.52	0.34	0.124	1.67 (0.87–3.23)

Note: JSS = Job Satisfaction Scale. The two profiles were dummy-coded: the “low well-being profile” was coded as 0 and the “high well-being profile” was coded as 1. * = *p* < 0.05 and *** = *p* < 0.001.

**Table 6:** Predictors of turnover intention.

Predictors	Turnover Intention (OTIS)			
	*b*	SE	*p*	OR (95% CI)
Pay (JSS)	−0.15	0.09	0.085	0.86 (0.72–1.02)
Promotion (JSS)	0.13	0.10	0.175	1.14 (0.94–1.34)
Supervision (JSS)	−0.30	0.07	<0.001	0.74 *** (0.64–0.86)
Fringe Benefits (JSS)	−0.22	0.11	0.048	0.80 * (0.65–0.99)
Contingent Rewards (JSS)	−0.10	0.09	0.272	0.90 (0.75–1.08)
Coworkers (JSS)	0.04	0.07	0.510	1.04 (0.92–1.19)
Nature of Work (JSS)	0.00	0.08	0.974	1.00 (0.86–1.17)
Communication (JSS)	0.18	0.08	0.019	1.20 * (1.03–1.40)
Work Safety	−0.82	0.40	0.037	0.44 * (0.20–0.95)
Work Shift Disorder	−0.59	0.60	0.331	0.55 (0.17–1.82)
Latent profile membership	−2.26	0.64	<0.001	0.10 *** (0.03–0.37)
Age	−0.12	0.03	<0.001	0.88 *** (0.82–0.95)

Note: JSS = Job Satisfaction Scale. The two profiles were dummy-coded: the “low well-being profile” was coded as 0 and the “high well-being profile” was coded as 1. * = *p* < 0.05 and *** = *p* < 0.001.

**Table 7:** Model fit statistics for path analysis models (Model 1 vs. Model 2).

Model	CFI	TLI	SRMR	RMSEA	90% CI for RMSEA
Model 1	1.00	1.03	0.02	0.00	0.00, 0.05
Model 2	1.00	1.02	0.02	0.00	0.00, 0.08

Note: Fit indices reported include CFI, TLI, RMSEA, and SRMR. Conventional thresholds: CFI/TLI ≥ 0.95, RMSEA ≤ 0.06, and SRMR ≤ 0.08.

## Data Availability

Data and materials of the current study are available in the Open Science Framework repository at the following link: https://osf.io/hazs3/overview?view_only=5917775441a84470a9ec42a9dd45a2eb, accessed on 4 March 2026.

## Supplementary Materials

The following supporting information can be downloaded at: https://www.mdpi.com/article/10.3390/ijerph23040485/s1, Table S1: Correlation matrix (Pearson) between the variables employed in the study.

## Abbreviations

The following abbreviations are used in this manuscript:

LPA	Latent Profile Analysis
JSS	Job Satisfaction Survey
POS	Perceived Occupational Stress scale
UWES-9	Utrecht Work Engagement Scale (short version)
WAMI	Work And Meaning Inventory
SWD	Shift work disorder
WSS	Work Safety Scale
OTIS	Ordinal Turnover Intention Scale
