# Supplementary material for: When Drivers Step Off the Bus: Well-Being and Turnover Intention in the Public Transport Sector
Source: Int J Environ Res Public Health. 2026 Apr 12;23(4):485. doi: 10.3390/ijerph23040485 (PMC13115743; doi:10.3390/ijerph23040485)
Supplement: Supplementary file 1 [file ijerph-23-00485-s001.zip › ijerph-4213626-supplementary.pdf]

## Supplementary Material A

**Table S1.** Correlation matrix (Pearson) between the variables employed in the study.

| Variable | 1 | 2 | 3 | 4 | 5 | 6 | 7 | 8 | 9 | 10 | 11 | 12 | 13 | 14 |
|---|---|---|---|---|---|---|---|---|---|---|---|---|---|---|
| 1. OTIS | — | | | | | | | | | | | | | |
| 2. JSS Pay | −.535*** | — | | | | | | | | | | | | |
| 3. JSS Promotion | −.423*** | .668*** | — | | | | | | | | | | | |
| 4. JSS Supervision | −.571*** | .547*** | .412*** | — | | | | | | | | | | |
| 5. JSS Fringe Benefits | −.517*** | .681*** | .703*** | .495*** | — | | | | | | | | | |
| 6. JSS Contingent Rewards | −.530*** | .731*** | .739*** | .634*** | .751*** | — | | | | | | | | |
| 7. JSS Coworkers | −.369*** | .412*** | .345*** | .507*** | .482*** | .541*** | — | | | | | | | |
| 8. JSS Nature of Work | −.513*** | .621*** | .533*** | .564*** | .546*** | .549*** | .291*** | — | | | | | | |
| 9. JSS Communication | −.410*** | .616*** | .565*** | .586*** | .709*** | .713*** | .559*** | .483*** | — | | | | | |
| 10. Work safety | −.419*** | .433*** | .479*** | .475*** | .553*** | .592*** | .521*** | .363*** | .592*** | — | | | | |
| 11. WAMI | −.554*** | .643*** | .673*** | .456*** | .609*** | .600*** | .304*** | .624*** | .511*** | .361*** | — | | | |
| 12. UWES-9 | −.581*** | .623*** | .559*** | .473*** | .618*** | .545*** | .273** | .736*** | .500*** | .483*** | .755*** | — | | |
| 13. POS | .391*** | −.498*** | −.427*** | −.564*** | −.540*** | −.502*** | −.507*** | −.495*** | −.588*** | −.716*** | −.322*** | −.473*** | — | |
| 14. Age | −.130 | −.152 | −.100 | −.126 | −.062 | −.117 | −.044 | −.116 | −.033 | −.127 | −.064 | −.149 | .265** | — |

Note. $p < .05$, p < .01, $p < .001$.
JSS = Job Satisfaction Survey; WSS = Work Safety Scale; WAMI = The Work and Meaning Inventory;
UWES-9 = Utrecht Work Engagement Scale; POS = Perceived Occupational Stress; OTIS = Ordinal Turnover Intention Scale.
